# Green Paradox or Forced Emission Reduction—The Dual Effects of Environmental Regulation on Carbon Emissions

**DOI:** 10.3390/ijerph191711058

**Published:** 2022-09-03

**Authors:** Kedong Yin, Lu Liu, Haolei Gu

**Affiliations:** 1 Institute of Marine Economy and Management, Shandong University of Finance and Economics, Jinan 250014, China; 2 School of Management Science and Engineering, Shandong University of Finance and Economics, Jinan 250014, China

**Keywords:** environmental regulation, carbon emissions, green paradox, double effect

## Abstract

In response to global climate change, China made a commitment about carbon emissions at the UN General Assembly. It will strive to achieve carbon peaking by 2030 and carbon neutrality by 2060. To help China successfully meet its carbon emissions targets this study examines the impact of environmental regulation on carbon emissions from a different perspective. Using panel data from 30 provinces in China as samples, this paper discusses the direct and indirect effect of environmental regulation on carbon emissions and explains the indirect process through four transmission paths: energy consumption structure, industrial structure, technological innovation, and foreign direct investment (FDI). The empirical results show that the direct effect of environmental regulation on carbon emissions presents an inverted U-shaped curve, it means that when the intensity level of environmental regulation is low, it mainly shows the green paradox effect, and with the continuous tightening of environmental laws, it turns into a forced emission reduction on carbon emissions. In addition, we found that under the constraint of environmental regulation conditions, the coal-based energy consumption is still the leading cause of carbon emissions; environmental regulations have contributed to the upgrading of industrial structure and technological advance, which indirectly play a positive role in carbon emission reduction. However, environmental regulation restrains the spillover effect and capital accumulation effect of FDI, which brings a specific degree of hindrance to technological progress and economic development, and is not conducive to carbon emission reduction. Therefore, we have made the following recommendations: China should make reasonable use of environmental policies to regulate carbon emissions according to the situation of each region, optimize the energy structure and increase the proportion of clean energy use, and improve the technology level of related industries to reduce carbon emissions by innovation.

## 1. Introduction

According to the IPCC (Intergovernmental Panel on Climate Change) report, the current global temperature has risen by 1.1 degrees Celsius over the pre-industrial era. If human beings continue to develop in a current way, the temperature will be 1.5 degrees Celsius higher than the pre-industrial level in the next 20 years [1], and at least 4 degrees Celsius by 2100. By that time, the polar ice sheet will melt, the sea level will rise by 0.1–0.9 m, flooding coastal land, causing global climate change, leading to extreme weather, frequent natural disasters, and various infectious diseases, which will cause great damage to the ecosystem, water and soil resources, human activities, and life safety [2]. China, as a country with 1/5 of the global population and high CO_2_ emissions (CO_2_ is the main component of greenhouse gases [3], is facing a more severe threat to people’s health and development challenges from global warming [4]. In response to climate change, China signed the Paris Climate Agreement in 2016 and pledged to the world at the United Nations General Assembly to achieve carbon peaking by 2030 and carbon neutrality by 2060 in 2020. The 14th Five-Year Plan (the 14th Five-Year Plan for National Economic and Social Development of the People’s Republic of China) also included “carbon peaking” as an important content, aiming to work with others country in the world to curb global warming, Climate change has become our most global challenge, and it is urgent to reduce carbon emissions.

CO_2_ emitted by human activities using carbon-based fossil energy is the leading cause of global warming [5]. Although China does not classify CO_2_ as a polluted gas, the United States, Japan, and other countries have passed legislation to determine CO_2_ as an air pollutant. Carbon emission is an external behavior in the process of production and consumption, and also belongs as an environmental resource. The market mechanism fails in regulating carbon emission, which needs third-party tools to restrain it [6]. Currently, most studies have verified that environmental regulation is an effective tool to regulate environmental and developmental issues [7], and therefore, it has become a consensus among governments to use environmental regulation tools to reasonably control carbon emissions. However, some scholars have put forward different views. Schou [8] argues that specific environmental policies are redundant. He believes that with the continuous growth and sustainability of the economy, the pollution level will naturally decline. The “Green Paradox” proposed by Sinn [9] also questions the validity of environmental laws on carbon emissions from another angle. In this way, it leads to a controversial issue that needs to be solved urgently: How exactly do environmental policies affect carbon emissions? The key to answering this question is to clarify the role and influencing process of environmental regulation on carbon emissions, which will be beneficial to the formulation and implementation of environmental laws in China, and have important guiding significance for China to achieve the goals of carbon emissions reduction.

Environmental regulation is a collection of legal policies designed to protect the environment. The government formulates and implements regulatory policies related to carbon emissions and intervenes in the economic activities of relevant financial entities, to regulate carbon emissions and thus achieving the purpose of reducing carbon emissions [10]. The effect of environmental policies on carbon emissions can be divided into two types: direct effect and indirect effect. Direct effects are mainly imposed through coercive measures (standards, command, and control) and financial instruments (subsidies, taxes, and fees, etc.,) [11]. The government adopts command-and-control instruments to carry out terminal treatment of carbon emissions. The main measures include shutting down some enterprises with high energy consumption and high emission, formulating emission standards, and forcing the use of clean energy [12]. On the other hand, uses taxes and fees or related incentive policies to manage at the source. By levying taxes on fossil energy producers and users and issuing environmental protection subsidies, enterprises are encouraged to reduce the demand for fossil energy and achieve the goal of reducing carbon emissions. Generally speaking, the objective of the government’s environmental policies is to protect the environment, achieve emission reduction while maintaining high quality economic development [13]. Hence, from an economic point of view, the environmental regulation will not completely implement the “one size fits all” model, thus leaving a specific room for the fossil material suppliers. The suppliers are worried that the future market will get worse and worse, and they will choose to move forward the mining path of related raw materials and increase the mining of related fossil raw materials, and put a large amount of fossil raw materials into the market. As a result, a large number of fossil fuels such as oil into the market will lead to a surge in supply, affect the relationship between supply and demand and prices, and prices will inevitably fall, eventually leading to an increase in consumption, resulting in an increasing pollution levels and carbon emissions. There is an effect contrary to the original intention of environmental policies—the green paradox proposed by Sinn [9].

The effect of environmental policies on carbon emissions not only has direct effects, but also may affect carbon emissions through other paths indirectly. By collecting relevant ecological policies and analyzing them, it is found that environmental regulation affects carbon emissions indirectly through four main pathways, namely raw materials, production, capital, and technology, which includes all the ways for environmental policy tools to play a role. Therefore, we studied the indirect effects of environmental regulation on carbon emissions from these four paths. First, environmental regulation can decarbonize the energy consumption structure of related industries by banning or limiting the use of fossil raw materials energy [14]. However, as mentioned above, such policies may have a green paradox effect, the more polluted the energy raw materials are, the more significant the impact of environmental regulations. Enterprises using such raw materials tend to increase their output to make up for the operating costs brought by environmental regulations, resulting in an increased carbon emissions in the short term [15]. Second, the implementation of environmental regulations will directly affect the production and development of related industries and influence the industrial structure of the region, while changes in the industrial structure will cause the level of carbon emissions in the region [16]. Third, for industries sensitive to environmental regulation, the degree of capital preference is more affected by environmental policies, such as manufacturing and extractive industries. The intensity of environmental regulation has a significant correlation with the ability to attract capital. Therefore, the level of environmental regulation indirectly contributes to carbon emissions by affecting the capital accumulation capacity of the relevant industries in the region [17]. Finally, environmental regulation can also indirectly affect carbon emissions by influencing the development of technology. Environmental regulation has both positive compensatory and negative crowding-out effects on technological development [18]. The positive compensation effect is that reasonable environmental policies can force enterprises to upgrade their management and technology, compensate for the “compliance costs” of complying with environmental regulation, and improve their productivity and competitiveness. The “crowding-out effect” refers to the fact that environmental regulation will increase the operating costs of enterprises, thus crowding out their R&D investment funds, which is detrimental to the advancement of technology in related industries [19]. The technological level of the relevant industry directly determines the level of productivity and carbon emissions of the industry. To sum up, the process by which environmental regulation acts on carbon emissions is complex. The root cause is that the implementation of environmental policies will have a multifaceted effect, and these changes directly or indirectly affect carbon emissions. To clarify the mechanism of the effects of environmental regulation on carbon emissions, in this study, the above pathways are analyzed empirically, and the influencing process of environmental regulation on carbon emissions is summarized, hoping to provide particular theoretical and practical support for China to achieve the goals of carbon peaking and carbon neutralization.

The main contributions of the research are as follows: First, the effects of environmental regulation on carbon emissions are divided into direct and indirect effects, and are analyzed empirically separately; second, the environmental regulation indicators are constructed from the cost of environmental regulation implementation and environmental tax revenue, respectively; and the environmental regulation indicators are incorporated into the model affecting carbon emissions to verify the trend of the impact of environmental regulation on carbon emissions; Third, the indirect effects of environmental regulation are grouped into four indirect transmission pathways: energy consumption structure, industrial structure, technological innovation, and FDI. By constructing a cross term between environmental regulation and the four influencing factors and incorporating the carbon emission impact model, the indirect effect mechanism of environmental regulation on carbon emissions is explored.

The structure of the paper is as follows: first, we analyze the process of environmental regulation on carbon emissions at the theoretical level. Second, we comb through the literature on the related fields involved and compile the latest progress of environmental regulation and carbon-emission-related research. Third, we construct a direct effect model and an indirect effect model of environmental regulation on carbon emissions, respectively, and explain the models and introduce the relevant statistical indicators and data sources. Fourth, we validate the collected data into the model to investigate how environmental regulation affects carbon emissions. Fifth, based on the empirical results and the theoretical analysis, we propose some substantive suggestions for reducing carbon emissions in China.

### Literature Review

Environmental regulation refers to the institutional arrangement by which the government restrains the emission behavior of economic agents by enacting administrative systems, using market mechanisms and playing the role of the public in order to regulate the failure of market mechanisms in environmental externalities and to pursue the goal of harmonious development of economy and nature [20]. As the only effective tool to regulate environmental resources, environmental regulation can directly affect the level of carbon emissions, but the mechanism of the effect of environmental regulation on carbon emissions is complex. On the one hand, environmental policies can directly control carbon emissions from the terminal, thus reducing carbon emissions, on the other hand, they can also indirectly increase the cost of enterprises, leading to the emergence of the green paradox effect. In order to better use environmental policies to control carbon emissions, it is crucial to clarify the mechanism of environmental regulations in carbon emissions.

As the only effective tool to regulate environmental resources, environmental regulation is an unavoidable topic in carbon emission research, and environmental regulation has been a hot topic in academic discussion. Researchers have conducted many interesting and meaningful studies on how environmental regulation affects carbon emissions. Generally speaking, environmental regulation is a set of laws for the protection of the environment, therefore, environmental regulation is supposed to play its proper role in regulating carbon emissions. Some scholars have verified this conclusion through experiments. For example, Neves [21] verified the effect of environmental laws of EU countries on CO_2_ emissions from a macro perspective, and believed that environmental regulation could effectively reduce carbon emissions. Zhang [22] also investigated the implementation effect of China’s environmental policies from the spatial dimension. Whether in the northeastern region with severe pollution or the southern provinces with relatively light pollution, the environmental regulation can effectively reduce carbon emissions to a certain extent. Du [23] evaluated the effect of environmental policies on pollution reduction and coordinated emission reduction from a micro perspective, taking enterprise data as samples. The experimental results show that environmental policies could significantly reduce CO_2_ emissions from China’s industrial sector. However, some people have put forward different views, among which Sinn’s “green paradox” effect has been recognized by considerable many scholars. Smulders [24] argued that imposing a carbon tax will lead to a “green paradox” effect, because it will stimulate the consumption of fossil energy in the transitional period, increasing carbon emissions. Van der Werf [25] agreed that some policies aimed at reducing greenhouse gas emissions may have the opposite effect. Zhang & Jia [26] used the panel data from Chinese cities as sample to explore and verify the relationship between environmental regulation and pollution emission in China, it is concluded that China still in the stage of the “green paradox.” Di Maria [27] also found that after the implementation of the Clean Air Amendment Act in 1990 in the United States, the price of coal dropped significantly, proving the existence of the green paradox effect from the side.

As mentioned above, the academic community has not yet reached a unified consensus on the effect of environmental regulation on carbon emissions, and we need to explore its mechanism of action in greater depth. Previously mentioned, the effects of environmental regulations on carbon emissions include direct and indirect effects, involving several factors, and the magnitude of the effects on carbon emissions varies in different contexts, which explains why academics hold different opinions on the direction of the effects of environmental regulations on carbon emissions. Therefore, we combed through the latest developments in related research. Among them, the direct effect of environmental regulation on carbon emissions has not shown much divergence, mainly because the direct effect is usually the end treatment of carbon emissions, and the government prohibits or limits carbon emissions through mandatory measures. The effect is obvious, most researchers agree that mandatory environmental policies have a significant effect on reducing carbon emissions [28,29,30]. However, there are other changes in objective conditions brought about by the implementation of environmental policies that in turn affect carbon emissions, and studies of such indirect effects have been proliferating, with researchers reaching different conclusions in different contexts. Concerning the path of “environmental regulation-energy consumption structure-carbon emissions,” Zhou [31] investigated the relationship between regional environmental policies and energy consumption structure in China, and the results show that environmental policies promoted technological progress and optimized energy consumption structure, but did not have much impact on emissions. Liu [14] also studied the relationship between regional environmental policies and energy consumption, and believed that the effect of different environmental laws on energy consumption could not be generalized, and it was impossible to determine the positive and negative relationship between the impact on carbon emissions. Wu [32] quantitatively examined the impact between energy consumption structure, environmental regulations, and carbon emissions and showed that the optimization of energy consumption structure has a significant contribution to carbon emission reduction, while the contribution increases as the intensity of environmental regulation increases. The researchers also found that environmental policies will influence the industrial development of the relevant regions, through optimizing the regional industrial structure and thus affecting the level of carbon emissions. Environmental policies can significantly increase the environmental protection costs of pollution-intensive industries, and enterprises meet environmental policies by shifting pollution sources [33], adjusting production and operation activities or increasing research and development investment in cleaner products [34,35], which will also change the regional industrial structure, which indirectly affects the carbon emission level of the region [36]. Chen [37] also proves that when the industrial structure is unreasonable, environmental regulation will promote CO_2_ emissions. When the structure is relatively reasonable, environmental regulation has a great effect on the suppression of CO_2_ emissions. Additionally, environmental policies can also affect the level of carbon emissions by influencing the degree of capital preference of the relevant industry, most directly in the sense that environmental regulations can significantly affect the level of Foreign Direct Investment (FDI) in the relevant industry, which has an indirect effect on carbon emissions. For example, Dong [38] takes the data of provincial cities in China as a sample. He verified that regions with relatively lax environmental regulation are more likely to attract investment, which is conducive to enhancing the development potential of related industries and can spread greener production technologies to host countries, bringing about a “pollution halo” effect. Dong [39] has revalidated this conclusion by analyzing the flow trend of FDI in China. The effect of environmental regulation on technological progress is also one of the ways to affect carbon emissions. Pei [40] analyzed the role of technical efficiency in bridging the gap between environmental regulation and carbon emission levels using provincial data from energy-intensive industries as a sample, and finally concluded that technical efficiency has a significant mediating effect between the environmental regulation and CO_2_ emissions. Wang [6] chooses a sample of 282 cities in China for empirical analysis, and the results show that there is an inverted U-shaped relationship between environmental regulation and carbon emissions, and in the long run, environmental regulation could effectively reduce carbon emissions by promoting industrial structure upgrading and technological advances.

Based on the above literature, it is not difficult to see that the mechanism of environmental regulation on carbon emissions is complex, and the conclusions of relevant academic research are also divergent. Moreover, most current studies focus on the single path of the effect mechanism of environmental regulation on emissions, which lacks scientificity and comprehensiveness. The effects of environmental regulation on carbon process are complex, and numerous factors affect diversity. In view of this, this paper further decomposes the relevant factors and examines the various ways in which environmental regulation affects carbon emissions by constructing a direct effect model and an indirect effect model, hoping to clarify the process and mechanism of environmental regulation on carbon emissions, so as to better answer the question of whether environmental regulation on carbon emissions is a green paradox or a forced emission reduction effect, and provide some theoretical and practical support for the formulation of carbon emission policies in China.

## 2. Materials and Methods

Since the direct effect of environmental regulation on CO_2_ emissions may not be a simple linear relationship, this paper introduces the quadratic term of environmental regulation (*ER*) to investigate the potential nonlinear implication. Based on these considerations, the following econometric models are constructed to measure the direct effects of environmental regulations on carbon emissions:

(1) $$C_{it}=\alpha_{1}ER_{it}+\alpha_{2}ER_{it}^{2}+\beta X_{it}+\alpha_{3}+\mu_{i}+\varepsilon_{\mathrm{it}}$$

The subscript *i* represents the region, *t* represents the year, *C* represents the CO_2_ emission of each region, *ER* represents the intensity of environmental regulation of each region, *X* represents other control variables except environmental regulation, $\alpha_{3}$ and $\mu_{i}$ represents the intercept term and random disturbance term of the model respectively, $\varepsilon_{\mathrm{it}}$ represents the error term of the model. 

In addition, to explore the indirect effect of environmental regulation on CO_2_ emission, we introduce the cross-items of environmental regulation (*ER*), energy consumption structure (*ES*), industrial structure (*Indu*), technological progress (*Tech*), and foreign direct investment (*FDI*) to investigate the mechanism and intensity of four ways to CO_2_ emission. The specific econometric models are as follows:

(2) $$\begin{array}{l} C_{it}=\alpha_{0}C_{it-1}+\alpha_{1}ER_{it}\times ES_{it}+\alpha_{2}ER_{it}\times {\mathrm{Indu}}_{it}+\alpha_{3}ER_{it}\times T{\mathrm{ech}}_{it}\\ \;+\alpha_{4}ER_{it}\times FDI_{it}+\beta X_{it}+\alpha_{3}+\mu_{i}+\varepsilon_{it} \end{array}$$

Among them, $ER_{\mathrm{it}}\times ES_{\mathrm{it}}$ represents the cross-item between environmental regulation and energy consumption structure in the *i* year of the *t* province; $ER_{\mathrm{it}}\times Indu_{\mathrm{it}}$ represents the cross-item between environmental regulation and industrial structure; $ER_{\mathrm{it}}\times Tech_{\mathrm{it}}$ represents the cross-item of environmental regulation and technical level; $ER_{\mathrm{it}}\times FDI_{\mathrm{it}}$ is the cross term of environmental regulation and *FDI*, and *X* is other control variables, including per capita income and population size. 

### 2.1. Carbon Dioxide Emissions (C)

Carbon emissions refer to the emissions of CO_2_ and other greenhouse gases. Considering that the proportion of CO_2_ in the global greenhouse gas clock is close to 80%, and it is more likely to cause climate warming than other greenhouses, most academic studies on carbon emissions only consider carbon dioxide [41]. CO_2_ emissions mainly come from fossil energy combustion and the industrial production of cement. According to the baseline method provided in the energy section of IPCC Guidelines for National Greenhouse Gas Inventories in 2006, the calculation formula of carbon dioxide emissions from fossil fuel consumption is as follows:

(3) $$EM=\sum EC_{i}\times CF_{i}$$

where *EM* represents the estimated total CO_2_ emissions of various energy consumption; *i* denotes the types of energy consumption. This paper selects nine primary fossil energy sources, including raw coal, cleaned coal, coke, crude oil, kerosene, gasoline, diesel oil, fuel oil, and natural gas; *EC_i_* is the consumption of type *i* energy (Available from the *China Energy Statistics Yearbook*.); *CF_i_* is the CO_2_ emission factor, and the calculation formula is as follows.

(4) $$CF_{i}=EQ_{i}\times EF_{i}\times COF_{i}\times T_{i}$$

Among them, *CF_i_* means the carbon emission coefficient of the *i* energy; *EQ_i_* is the low calorific value, *EF_i_* is the carbon emissions factor, *COF_i_* is the carbon-oxygen conversion rate, and *T_i_* is the carbon conversion coefficient, which is usually 12/44. The relevant data of IPCC National Greenhouse Gas Emission Inventory Guidelines are selected, specifically raw coal 26.8, washed coal 25.8, coke 29.2, crude oil 20, kerosene 19.5, gasoline 18.9, diesel oil 20.2, fuel oil 21.1 and natural gas 15.3, all in TC/TJ. 

The CO_2_ emission formula of the cement production process is

(5) CC=Q×β

where *CC* represents the total amount of CO_2_ emission in the cement production process, *Q* represents the total amount of cement production, β represents the CO_2_ emission coefficient in the cement production process clock. 

The final calculation formula of total CO_2_ emission is:

(6) C=EM+CC

### 2.2. Environmental Regulation (ER)

Currently, the academic community has not yet developed an authoritative approach for the measurement of environmental regulation. Most researchers choose different measurement methods according to the research objects. This study focuses on the effect of environmental regulation on greenhouse gas control, and we decide to use the payment cost of pollution control to express the intensity of environmental regulation [42]. Considering the actual situation in China, the payment cost of pollution control is mainly composed of three parts, which are investment in pollution treatment equipment, depreciation of pollution treatment equipment and operating cost of pollution treatment equipment. According to the statistical way in China, we find that the investment amount and depreciation cost of pollution treatment equipment can be expressed by the investment amount of pollution treatment. Operating cost of pollution treatment equipment refers to the enterprise in order to deal with the pollutants generated in the production process, need to be equipped with professional pollution treatment facilities, the cost incurred in the operation of the facilities is the operating cost of pollution treatment equipment. This indicator could more intuitively reflect the level of local environmental regulation intensity. In China’s statistical approach, pollution equipment operating costs are mainly divided into waste gas and waste water treatment facilities operating costs, and the main object of this paper is air pollutants, so we choose waste gas treatment equipment operating costs as an indicator to measure the level of environmental regulation. Therefore, we choose the investment amount of pollution control and the operation cost of waste gas treatment facilities to indicate the payment cost of pollution control.

Although the payment cost of pollution control can better reflect the level of environmental regulation in a region, this paper takes provincial-level regions as the research sample, and due to the large development gap and different development patterns among regions in China, the absolute value of pollution control payment cost alone cannot eliminate the differences caused by the development level of each region. Therefore, we consider using the pollution treatment payment cost per unit of industrial output value to indicate the level of environmental regulation in the region, and to avoid the environmental cost advantage of each region, we also adopt the relative level of environmental regulation proposed by Peng et al. [43] to measure environmental regulation, which refers to the relative level of environmental regulation between the pollution treatment payment cost per unit of industrial output value in each region and the national. The ratio of the cost of pollution treatment paid per unit of industrial output value in each region to the cost of pollution treatment paid per unit of industrial output value is given by

(7) $$PC_{\mathrm{it}}=\frac{PI_{i}{}_{t}+IF_{\mathrm{it}}}{IGDP_{\mathrm{it}}}$$

(8) $$ER_{\mathrm{it}}=PC_{\mathrm{it}}/\frac{\sum PI_{i}{}_{t}+IF_{\mathrm{it}}+}{\sum IGDP_{\mathrm{it}}}$$

Among them, *PI* and *IF* respectively represent the investment amount of pollution control and the operation cost of waste gas treatment facilities, *PC* represents the payment cost of pollution control of the industrial unit output value of each province, *IGDP* represents the gross industrial output value of each region, and *ER* represents the relative environmental regulation level of each region. This indicator is a relative value, which not only eliminates the differences caused by the level of development between different regions, but also avoids the errors caused by environmental advantages. The indicator is non-negative, and a value of 1 indicates that it reaches the national average; the larger the value, the greater the intensity of environmental regulation.

### 2.3. Other Variables

To measure the impact of environmental regulation on carbon emissions, other variables need to be controlled, such as different economic structures, social factors, etc. This paper selects the following control variables.

(1) Per capita gross domestic product (PGDP). To some extent, per capita GDP can reflect the development level of a region. The better the economic development, the greater the carbon emissions, so the prediction coefficient of this index is positive.

(2) Energy consumption structure (*Energy*). The indicator can reflect the economic composition structure of the region to some extent. We express it in terms of the share of coal consumption in total energy consumption. If the energy consumption is primarily carbon energy, the carbon emissions in this region are correspondingly higher, and if the energy consumption is primarily clean energy, the carbon emissions are lower. Therefore, the prediction coefficient of this index is positive.

(3) Industrial structure (*Industry*). This indicator mainly examines the share of the secondary industry in the region. The secondary industry is usually industry and manufacturing, and most of them belong to carbon emission-intensive sectors. We select proportion of the total output value of the secondary industry to the total output value of this region. The indicator reflects the industrial structure of an area. The high ratio of secondary industry means that the area is dominated by industrial sectors and more intensive carbon emissions. Hence the prediction coefficient of this index is positive.

(4) Technology (*Tech*). The technical level can directly affect a region’s economic structure, production level, and pollution emission during the production process. We choose the ratio of R&D expenditure to GDP of each province to measure it. Generally speaking, the lower the carbon emission level in the region with a relatively high technical level, so the prediction coefficient of this index is negative.

(5) Foreign direct investment (*FDI*). This indicator can directly measure a region’s ability to attract capital. This paper uses the proportion of actual foreign direct investment to GDP for calculation. Usually, worth tends to those regions with low human resource costs. In contrast such areas are generally relatively backward and have relatively high carbon emissions, so the index prediction coefficient is changed to positive.

(6) Population size (*Pop*). Population size is a basic indicator of a region and occupies an important position in studies about environment and economy. Population size determines the size of carbon emissions to some extent and is a key indicator of carbon emission level. Generally speaking, the higher the number of populations, the higher the demand of carbon emission, and the prediction coefficient of this indicator is positive.

This paper selects panel data from 30 provinces and cities (except Tibet) in China from 2004 to 2019 for the empirical test. The above data mainly come from China Statistical Yearbook, China Regional Economic Statistical Yearbook, China Energy Statistical Yearbook, China Environmental Statistical Yearbook, and so on. Due to the existence of inflation factors, the indicators related to the price index are adjusted to constant prices based on 2000, and the statistical description of all variables is shown in Table 1. 

## 3. Results

### 3.1. The Direct Effect of Environmental Regulation on Carbon Emissions

Hausman test results show that the equation rejects the original hypothesis at 1% significance level, so we use fixed effect model to fit the original data. Different control variables are added to models (1)–(6) to observe the stability of the environmental efficiency level coefficient, and the estimated results are shown in Table 2.

This paper adopts the method of empirical analysis by gradually adding variables. From Table 2, it can be seen that the primary coefficients of environmental regulation level are significantly positive and the secondary coefficients are significantly negative, which means that the effect of environmental regulation on CO_2_ emission presents an inverted U-shaped curve, meaning when the environmental regulation level is low, there will be a “green paradox” phenomenon, and then with the enhancement of environmental regulation intensity, after reaching a certain threshold, it can play the role of “forcing emission reduction”. The reasons for this phenomenon can be attributed to the following: At the initial stage of the implementation of environmental regulation, the relevant policies need to be improved, which leads some enterprises to move forward on the mining path of fossil energy in order to avoid the compliance cost of environmental regulation and maximize profits, which makes the CO_2_ emission increase in the short term, showing the rising stage of inverted U-shaped curve. With the increasing environmental pressure and the continuous improvement of environmental regulation level and enforcement, the passive acceptance of environmental regulation brings about the rising operating costs of enterprises. Faced with this situation, related enterprises will actively increase investment, improve production technology, optimize enterprise operation methods and other operational efficiencies for long-term development, so as to make up for the rising production and operating costs and force enterprises to upgrade production and innovate technology. At the same time, in order to meet the requirements of relevant environmental policies, it will promote the use of clean energy and the progress of green technology and truly realize the forced emission reduction effect of environmental regulation. To sum up, the impact of environmental regulation on carbon emissions is not a simple linear relationship but presents an inverted U-shaped curve under the action of many factors. The initial stage is a green paradox stage, and the later stage can promote the upgrading of related industries and the development of technology while realizing emission reduction. Therefore, environmental regulation plays an active role as a policy tool to adjust the economy and environment. According to the results of model (6), combined with the primary term and quadratic form of environmental regulation level, it is found that the inflection point of environmental regulation on carbon emissions is 1.83, that is to say, when the environmental regulation level of a region exceeds the national average level by 0.8 times, environmental regulation begins to play a role in reducing carbon emissions, and with the improvement of environmental regulation level, carbon emissions will gradually decrease.

### 3.2. Indirect Effects of Environmental Regulation on Carbon Emissions

This paper studies the indirect effects of environmental regulation on carbon emissions by introducing the cross-items of environmental regulation, foreign direct investment, energy consumption structure, industrial structure, and technological innovation level. Through Hausman test, the fixed effect model is still used, and the related variables are regressed, respectively.

As can be seen from Table 3, environmental regulation has indirect effect on carbon emissions through four different paths. (1) Under the constraints of environmental regulations, FDI has a significant positive impact on carbon emissions. The main reason for this phenomenon is that strict environmental regulation will significantly increase the operating costs of foreign-funded enterprises, raise the entry threshold of foreign-funded enterprises, and is not conducive to absorbing the technology and capital of foreign-funded excellent enterprises, thus weakening the spillover effect of FDI. In addition, due to the “pollution refuge” effect, the intensity of environmental regulation is enhanced, which makes foreign-funded enterprises flee to areas with low intensity of environmental regulation, thus weakening the regional capital stock, which is not conducive to economic development and technological progress. Generally speaking, environmental regulation indirectly hinders the level of regional technological innovation and has a negative impact on carbon emissions by restraining the spillover effect of FDI and capital stock. (2) Environmental regulation indirectly affects carbon emissions through the energy consumption structure of relevant regions. This phenomenon is mainly due to the fact that China is a typical country with more coal and less oil. Although environmental regulation will effectively curb carbon emissions in theory, according to statistics, China has not yet achieved low-carbon development, and fossil energy such as coal is still the leading energy in China at present. It still has a long way to go to reduce carbon emissions by forcing low-carbon development through environmental regulation. (3) Under the constraint of environmental regulation, the influence of industrial structure on carbon emissions is significantly negative, that is, environmental regulation can reduce carbon emissions by adjusting industrial structure. The main reason is that the promotion of environmental regulation increases the operating costs of industrial industries, thus improving the survival threshold of related enterprises, inhibiting the secondary industry, promoting the optimization of industrial structure, and indirectly reducing energy consumption and carbon emissions. (4) The cross-item between environmental regulation and technological innovation is significantly negative, which shows that technological innovation has a positive effect on carbon emission reduction under the constraint of environmental regulation, which is consistent with the previous introduction. Environmental regulation will force enterprises to reform, promote technological innovation and technological progress; this will improve the operational efficiency of enterprises, and at the same time reduce environmental pollution, which plays a positive role in carbon emission reduction.

## 4. Robustness Test

This section chooses another method to measure the level of environmental regulation, that is, the total amount of taxes and fees collected [44,45] measures the intensity of environmental regulation. We choose sewage charges per unit industrial output value (changed to environmental protection tax after 2018) to express the level of environmental regulation. The data come from China Environmental Statistics Yearbook and China Tax Yearbook, etc. We also analyze the direct and indirect effects of environmental regulation on carbon emissions and test the robustness of the above analysis conclusions. The test results are shown in Table 4. It is not difficult to see that the direct effect of environmental regulation on carbon emissions still shows a significant inverted U-shaped relationship, and the indirect effect on carbon emissions is also significant through four transmission paths, which is consistent with the conclusion in Table 3, which shows that our conclusion is robust.

## 5. Conclusions

Is the impact of environmental regulation on carbon emissions a green paradox or a forced emission reduction effect? Through the analysis and verification presented in this paper, it was found that the impact mechanism of environmental regulation on carbon emissions is complex, which depends not only on the level of environmental regulation but also on various objective factors. Generally speaking, the impact of environmental regulation on carbon emissions presents an inverted “U” curve, that is, the effect of environmental regulation on carbon emissions is not a simple linear relationship but a parabola. When the intensity of environmental regulation is low, the impact on carbon emissions mainly shows the green paradox effect. After reaching a certain threshold, it shows the reverse emission reduction effect. As far as China’s actual situation is concerned, the level of environmental regulation still fails to change the current consumption structure with coal as the primary energy, which leads to the low effect and income of carbon emission reduction policy. According to our calculation, the intensity of environmental regulation in China still needs to be nearly doubled before it can bring better carbon emission reduction effect. On the other hand, the implementation of China’s environmental policies has also promoted technological innovation and technological progress, and it has a positive effect on the optimization of industrial structure, laying a foundation for the realization of carbon peaking and carbon neutralization goals in the future to a certain extent. However, at the same time, the implementation of environmental regulation also restricts the entry of foreign capital to a certain extent, thus inhibiting the development of local related industries.

However, there are some shortcomings in this study: (1) the method of measuring the level of environmental regulation is still controversial, and although this paper chooses a measure with high academic recognition, the measurement of environmental regulation is still a complex process, which cannot accurately reflect the strength of environmental regulation, which may bring some negative impact on the results. (2) In this paper, we choose panel data from 30 provinces in China, but there are some missing data in different years in each province, although we use different methods to complete them, but still, it will cause some errors. In summary, the relationship between environmental regulations and carbon emissions is intricate and complex. Whether it is a green paradox effect or a carbon reduction effect needs to be studied separately according to different regions, which well explains why the studies of different scholars in the literature review section present different conclusions. Unlike previous studies, this paper focuses on the relationship between environmental regulations and carbon emissions, using Chinese regional panel data as a sample, and finds that the relationship between the two is not simply linear, but environmental regulations show different effects in different periods. In addition, by combing previous studies, this paper summarizes the indirect action pathways of environmental regulations on carbon emissions, which reveals the direct and indirect effects of environmental regulations on carbon emissions in a more comprehensive way and makes a certain contribution to the study of the mechanism of the effect of environmental regulations on carbon emissions.

## 6. Recommendations

Through the analysis of the correlation between environmental regulation and carbon emissions, this paper clarifies the mechanism of policy tools in regulating carbon emissions and it gives the impact direction of environmental regulation on carbon emissions from direct effect and indirect effect, which provides a theoretical reference for helping to achieve China’s dual-carbon goal. In view of this, the following relevant policy suggestions are given: (1) strengthen the intensity of environmental regulation reasonably. As an effective policy tool to regulate the environment and economy, the rational use of environmental regulation is an important prerequisite to achieving the goal of carbon neutrality in China. Strengthening environmental regulation level is not only conducive to carbon emission reduction but also promotes industrial upgrading and technological progress to a certain extent. However, the use of environmental regulations should not be blind, so as not to hinder economic development. (2) Optimize the energy consumption structure. Vigorously develop clean energy such as wind and tidal energy, increase the promotion of clean energy, and build an energy consumption structure based on clean energy. (3) Developing high-end science and technology, increasing investment in scientific and technological research and development, innovation is still the main driving force of social development. Technological progress can not only effectively promote carbon emission reduction but also liberate and enhance productivity. Establishing a perfect scientific research guarantee system and innovation incentive policies is the principal strategic starting point for achieving a win-win situation in environmental and economic development. (4) Optimize FDI utilization strategy and enhance FDI spillover effect. Although environmental regulations hinder the inflow of foreign capital to a certain extent, it does not mean that environmental regulations and FDI are incompatible. According to the regional situation, we should reasonably formulate environmental policies, increase the attraction of high-quality FDI, eliminate the introduction of low-quality FDI, avoid becoming the pollution refuge of developed countries, strengthen the guiding role of the government in the introduction of foreign capital, optimize the level of FDI utilization, and thus increase the environmental spillover effect of FDI. (5) Improve environmental technology research and development. The level of carbon emissions is closely related to regional economic development, and simply suppressing carbon emissions will harm the development of the economy, reduce the level of carbon emissions in the process of economic development by improving environmental protection technology, increase the investment in environmental protection technology R&D, improve the ability of independent innovation, and remove the obstacles caused by carbon emissions in the process of economic development.

## Figures and Tables

**Table 1:** Descriptive statistics.

Name	Symbol	Mean	Std.Dev	Min	Max	Predictive Coefficient
CO_2_ emissions	lnC	5.434	0.827	2.022	7.438	/
Environment regulation	ER	1.863	2.648	0.541	9.169	?
Energy structure	Energy	0.677	0.284	0.017	1.758	+
Industrial structure	Industry	0.435	0.827	0.162	0.620	+
Technology innovation	Tech	1.460	1.077	0.180	6.31	−
Foreign direct investment	FDI	0.024	0.022	0.0001	0.136	+
GDP per capital	lnPgdp	10.345	0.685	8.353	11.994	+
population	lnPop	8.177	0.750	6.289	9.433	+

Note: “+” means positive; “−” means negative; “/” means no predictive coefficient.

**Table 2:** Direct effects of environmental regulation on carbon emissions.

Model	(1)	(2)	(3)	(4)	(5)	(6)
*C*	−0.020 (0.178)	−1.007 *** (0.169)	−1.013 *** (0.219)	−1.415 *** (0.220)	−1.413 *** (0.221)	−7.083 *** (1.480)
*ER*	0.086 *** (0.018)	0.031 * (0.016)	0.033 ** (0.015)	0.033 ** (0.015)	0.033 ** (0.015)	0.033 ** (0.016)
*ER2*	−0.025 *** (0.006)	−0.007 * (0.004)	−0.008 * (0.005)	−0.007 * (0.005)	−0.008 * (0.005)	−0.009 * (0.005)
*Lnpgdp*	0.514 * (0.016)	0.537 *** (0.014)	0.472 ** (0.017)	0.561 *** (0.018)	0.556 *** (0.019)	0.547482 *** (0.019)
*Energy*		1.217 *** (0.092)	1.176 * (0.099)	1.186 *** (0.098)	1.186 *** (0.097)	1.200 *** (0.096)
*Industry*			0.004 *** (0.002)	0.005 *** (0.002)	0.005 *** (0.002)	0.006 *** (0.002)
*Technology*				−0.001 (0.035)	−0.001 (0.035)	−0.072 * (0.039)
*FDI*					−0.075 (0.659)	0.055 (0.650)
*Lnpop*						0.712868 *** (0.184)
*Fixed time*	yes	yes	yes	yes	yes	yes
*Fixed region*	yes	yes	yes	yes	yes	yes
*ADJ-R2*	0.58	0.621	0.681	0.729	0.774	0.782
*Hausman test*	29.83	32.15	30.17	29.15	28.97	35.02
*p-Value*	0.00	0.00	0.00	0.00	0.00	0.00

Note: Standard errors are in parentheses; *, **, and *** indicate significance at the 10%, 5%, and 1% levels, respectively.

**Table 3:** Indirect effects of environmental regulation on carbon emissions.

Model	(1)	(2)	(3)	(4)
*C*	−0.426 (1.528)	0.074 (1.504)	0.315 (1.438)	0.562 (1.441)
*ER × FDI*	0.837 ** (0.409)	0.109 (0.436)	0.515 (0.421)	0.692 * (0.432)
*ER × Energy*		0.052 *** (0.012)	0.242 *** (0.313)	0.231 *** (0.031)
*ER × Industry*			−0.0029 *** (0.001)	−0.003 *** (0.0004)
*ER × Tech*				−0.024 * (0.013)
*lnpgdp*	0.507 ** (0.200)	0.507 *** (0.196)	0.484 ** (0.019)	0.474 ** (0.197)
*lnpop*	0.071 * (0.019)	0.056 * (0.019)	0.055 * (0.018)	0.054 * (0.011)
*ADJ-R2*	0.461	0.481	0.513	0.729
*Hausman test*	12.09	15.77	17.24	15.09
*p-Value*	0.00	0.00	0.00	0.00

Note: standard errors are in parentheses; *, **, *** indicate significant at the level of 10%, 5%, and 1%, respectively.

**Table 4:** Robustness test.

Dependent Variable	Direct Effect		Dependent Variable	Indirect Effect	
	Coefficient	*t*-Value		Coefficient	*t*-Value
*C*	0.231 *	2.865	*C*	0.790	6.217
*ER*	0.142 *	4.901	*ER × FDI*	0.002 **	−1.208
*ER2*	−0.031 **	−0.134	*ER × Energy*	0.001 *	2.334
*Lnpgdp*	0.474 ***	3.127	*ER × Industry*	−0.002 **	1.458
*Energy*	3.108 **	8.900	*ER × Tech*	−0.099	−3.210
*Industry*	0.001 *	−1.235	*lnpgdp*	1.295	8.357
*Technology*	−0.295 ***	8.647	*lnpop*	0.139 *	2.121
*FDI*	0.0989	7.661			
*Lnpop*	0.020 *	−2.001			
*ADJ-R2*	0.549		*ADJ-R2*	0.626	
*Hausman test*	18.22		*Hausman test*	21.90	
*p-Value*	0.00		*p-Value*	0.00	

Note: Standard errors are in parentheses; *, **, *** indicate significant at the level of 10%, 5%, and 1%, respectively.

## Data Availability

The data presented in this study are available on request from the corresponding author. The data are not publicly available due to legal and privacy issues.
